# Chromium oxide coatings with the potential for eliminating the risk of chromium ion release in orthopaedic implants

**DOI:** 10.1098/rsos.170218

**Published:** 2017-07-05

**Authors:** A. M. Oje, A. A. Ogwu

**Affiliations:** School of Engineering and Computing, University of the West of Scotland, High Street, Paisley PA1 2BE, UK

**Keywords:** corrosion, chromium oxide coating, adhesion strength, Ringer's solution, medical implant

## Abstract

Chromium oxide coatings prepared by radiofrequency reactive magnetron sputtering on stainless steel substrates were exposed to Ringer's physiological solution and tested for their electrochemical corrosion stability using an open circuit potential measurement, potentiodynamic polarization, electrochemical impedance spectroscopy and Mott–Schottky analysis. The coatings were found to be predominantly Cr_2_O_3_, based on the observation of the dominance of ${\text{A}}_{1_{g}}$ and E_g_ symmetric modes in our Raman spectroscopic investigation and the E_u_ vibrational modes in our Fourier transform infrared spectroscopic measurements on the coatings. We investigated for the presence of chromium ions in Ringer's solution after all of the above electrochemical tests using atomic absorption spectroscopy, without finding a trace of chromium ions at the ppm level for coatings tested under open circuit and at the lower potentials implants are likely to experience in the human body. The coatings were further exposed to Ringer's solution for one month and tested for adhesion strength changes, and we found that they retained substantial adhesion to the substrates. We expect this finding to be significant for future orthopaedic implants where chromium ion release is still a major challenge.

## 1. Introduction

One of the major challenges faced by patients with metal-on-metal hip replacements is the potential of implant failure. The implant failure *in vivo* is well known to involve corrosion, which leads to the release of wear debris/ions and have been linked with possible adverse health effects such as pains, pseudo-tumour formation and inflammation in patients [1–3]. Materials such as titanium alloys, 316 L stainless steel, cobalt-based alloys, zirconium alloy, ultra-high molecular weight polyethylene and alumina-based ceramics are widely used as implant materials due to their high corrosion resistance, mechanical properties and biocompatibility. However, the above-mentioned implant materials are not immune to wear, corrosion and biocompatibility issues. Studies conducted on retrieved 316 L stainless steel implant show that more than 90% of failures of the material is due to pitting and crevice corrosion attack [4], resulting in the release of Fe, Cr and Ni ions, which are toxic to the human body system. Nevertheless, its low cost and poor corrosion resistance compared with other biomaterials (like cobalt chromium molybdenum and titanium alloy) make it suitable for temporary implants like screws and plates that are normally removed after a few years. Cobalt chromium molybdenum alloy (CoCrMo) implant, on the other hand, releases cobalt and chromium ions into the surrounding tissue fluid because of wear at the bearing surface and the corrosion of the implant material [5–7]. The biocompatibility of an implant material is strongly linked to its resistance to tribological and corrosion processes. These properties can be enhanced by applying a wear and corrosion protective coating material on the implant surface to eliminate or minimize surface damage and degradation while retaining the bulk material properties.

Several coating materials such as diamond-like carbon (DLC), chromium nitride (CrN), hydroxyapatite (HA), titanium nitride (TiN) and titanium niobium nitride (TiNbN) [8–14] have been explored by different researchers for orthopaedic implant applications. While some of these coatings are either in their early or advanced stages of investigation, others such as TiN and TiNbN are already in use in commercially available hip and knee prostheses [13,14]. One of the major drawbacks for some of these coatings is delamination, which occurs due to poor adhesion on substrates resulting from the high level of internal stress generated during the coating process. Contrary to the excellent results obtained from *in vitro* wear simulator studies on DLC coatings, a poor adhesion of the coatings on the substrate was reported during *in vivo* tests [15,16]. Raimondi *et al.* [17] reported a local delamination and coating breakdown on case studies of explanted TiNi-coated hip implants. HA coatings, on the other hand, despite their excellent biomaterial properties have inherent mechanical properties such as poor tensile strength, poor impact resistance and brittleness that have restricted their use for many load-bearing applications [18]. Chromium oxide coatings can be prepared using various techniques such as sputtering, chemical vapour deposition, electron-beam evaporation, plasma spray pyrolysis, arc ion plating and pulse laser deposition [19–25]. Of all the deposition techniques, radiofrequency reactive magnetron sputtering is regarded as the most preferable for industrial production as this technique will enable the synthesis of high-quality Cr_2_O_3_ with desired hardness and wear properties. Previous studies on chromium oxide coatings prepared by magnetron sputtering have revealed that, depending on the preparation method and deposition conditions used during the deposition, films with single or mixed phases and varying properties can be formed [19,26,27]. Barshilia & Rajam [26] and Hones *et al.* [19] reported the presence of single-phase Cr_2_O_3_ for the chromium oxide coating prepared using pulsed DC reactive unbalanced magnetron sputtering and reactive magnetron sputtering while films with mixed phases of Cr_2_O_3_ and CrO_3_ were obtained by Pang & Gao [27] using reactive unbalanced magnetron sputtering. The authors observed a maximum hardness value of 22 GPa for films prepared by pulse DC unbalanced magnetron sputtering and a hardness of 32 GPa for films prepared by reactive magnetron sputtering [19,26]. A number of investigations conducted on the corrosion properties of chromium oxide coatings have shown that chromium oxide coatings possess good corrosion resistance in phosphate-buffered saline (PBS) solution and saline solution [26,28–30], and have the capability to act as a protective coating material for stainless steel and other substrates exposed to the aforementioned solutions. It has been shown that chromium oxide coatings prepared by radiofrequency reactive magnetron sputtering, which are predominately single-phase Cr_2_O_3_, release a minimal amount of chromium ions during electrochemical tests in saline solution [29]. The authors reported that the ion release behaviour of the coating is most probably due to the lower defect density on the surface of the coatings. In order for chromium oxide coatings to be considered a viable candidate material for implant applications, their electrochemical behaviour in physiological solutions such as Ringer's and Hanks’ solutions, which contain ion concentrations close to those of the human body, needs to be investigated.

We now report our investigation of the corrosion and adhesion behaviour of chromium oxide coatings prepared by reactive magnetron sputtering when exposed to Ringer's solution. This is necessary for evaluating their suitability for implant applications.

## 2. Theory of the substrate plastic straining adhesion test

The substrate plastic straining test is an adaptation based on the shear-lag analysis for composites as shown below. Cox [31] using the shear-lag analysis has shown that for a fibre of length *L* embedded in a matrix subjected to strain, as long as the fibre is sufficiently long, the stress in the fibre will increase from the two ends to a maximum value and the average stress in the fibre will be given by

2.1 $$\sigma_{f}=E_{f}\cdot \text{e}\;\left[1-\frac{\tanh(\beta L/2)}{\left(\beta L/2\right)}\right],$$

where

2.2 $$\beta =\left[\frac{2Gm}{E_{f}r^{2}\ln(R/r)}\right],$$

Here, *e* is the strain, *Gm* is the shear modulus of matrix, *E*_f_ is the fibre elastic modulus, *L* is the fibre length and *R* is the distance between neighbouring fibres.

The variation in the shear stress along the fibre–matrix interface can be expressed as

2.3 $$\tau =\frac{E_{f}.r_{f}.e.\beta }{2}\cdot \frac{\sinh\beta [(L/2)-x]}{\text{cosh}\beta (L/2)},$$

where *x* is the distance from one end of the fibre and *r*_f_ is the fibre radius.

Kelly & Tyson [32] further proposed a relationship for the interfacial shear strength in the above situation, given by

2.4 $$\tau =\frac{\sigma_{\mathrm{fu}}.d}{2L_{c}},$$

where *d* is the fibre diameter, *L*_c_ is the critical fibre length and *σ*_fu_ is the fibre strength at a length equal to *L*_c_.

As a range of values will occur for the critical fibre length, *L*_c_, a set of proposals have been made for determining the *L*_c_ value to be used in equation (2.4) using fibre length mean values obtained by fitting the experimental fibre length distribution measurements to the most closely matching probability distribution of fibre lengths, e.g. Weibull, lognormal, etc.

The substrate-straining technique is a variant of the method for determining the interfacial shear strength for fibre-reinforced composites developed by Kelly & Tyson [32] in equation (2.4). A non-ductile thin film/coating deposited on a ductile substrate is subjected to a tensile stress and deformed plastically. The strain at which the cracks begin to appear on the film/coating, observed by scanning electron microscopy, gives a measure of the tensile fracture strength of the film, expressed as [33]

2.5 $$\sigma =\varepsilon_{f}.E,$$

where *σ* is the film fracture stress, *E* is Young's modulus of the film and *ϵ*_f_ is the fracture strain.

The substrate–film system is then continuously strained, and the spacing of the cracks on the film is monitored by the observation of the test specimen in the scanning electron microscope at various strains, until saturation is achieved, i.e. a steady-state spacing of cracks. The crack spacing distribution under steady-state conditions is then determined and the mean crack spacing *λ* is evaluated. The interfacial shear strength is determined by substituting *λ* into the following equation:

2.6 $$\tau =\frac{\sigma .\delta }{\lambda },$$

where *σ* is the film fracture stress, *δ* is the film thickness of the film and *λ* is the average crack spacing at saturation.

## 3. Mott–Schottky analysis

Mott–Schottky analysis was conducted to determine the electronic defect properties of oxide layers formed on the surface of the chromium oxide-coated and -uncoated stainless steel substrates. The relationship between the capacitance and the applied potential is given by the well-known Mott–Schottky equations for *n*-type and *p*-type semiconductors and are shown in equations (3.1) and (3.2), respectively:

3.1 $$C^{-2}=\frac{2}{\varepsilon \varepsilon_{o}QN_{D}}\left(E-E_{\mathrm{FB}}-\frac{KT}{Q}\right)$$

and

3.2 $$C^{-2}=-\frac{2}{\varepsilon \varepsilon_{o}QN_{A}}\left(E-E_{\mathrm{FB}}-\frac{KT}{Q}\right),$$

where *ϵ_o_* is the permittivity of free space (8.85 × 10^−14^ F cm^−1^), *ϵ* is the dielectric constant of the passive film, *Q* is the electron charge (1.602 × 10^−19^ C), *N*_D_ and *N*_A_ are the donor and acceptor density, respectively, *E*_FB_ is the flat-band potential, *K* is the Boltzmann constant (1.38 × 10^−23^ J K^−1^) and *T* is the absolute temperature. We see that *N*_D_ and *N*_A_ can be determined from the slope of the experimental *C*^−2^ versus applied potential (*E*), while the intercept on the potential axis corresponds to the flat-band potential *E*_FB_. The validity of Mott–Schottky analysis is based on the assumption that the capacitance of the space charge layer is much smaller than the double-layer capacitance.

## 4. Experimental methods

### 4.1. Film deposition

Chromium oxide thin film deposition was carried out with a cryo-pumped vacuum chamber radiofrequency magnetron sputtering unit using the deposition conditions given in table 1. A solid chromium target was used as the starting material with high-purity argon and oxygen as sputtering and reactive gases, respectively. The substrate materials were glass slides, silicon wafer and 304 medical grade stainless steel. Each of the steel substrates for tensile tests were machined into a dog bone shape for mechanical testing and the substrates for corrosion testing were cut to a square shape of 80 × 80 mm, ground and metallographically polished. The substrates were cleaned ultrasonically with isopropanol and then washed with de-ionized water. Prior to deposition, the deposition chamber was evacuated to a pressure of about 8 µTorr.

**Table 1.** Deposition conditions for the prepared chromium oxide thin films.

forward power	300, 350, 400, 450 and 500 W
oxygen flow rates	2, 4, 6, 8 and 10 sccm
argon flow rate	60 sccm
deposition time	120 min
substrate temperature	ambient temperature
reflected power	<6 W

### 4.2. Material characterization

The chemical constituents of the prepared chromium oxide films were probed with Raman spectroscopy, Fourier transform infrared (FTIR) spectroscopy and X-ray photoelectron spectroscopy (XPS). A Thermo Scientific DXR Raman microscope, Nicolet iS50 FTIR equipment and Scienta ESCA 300 spectrophotometer equipped with a monochromatic Al Kα (1486.6 eV) X-ray source were used for the measurements. The XPS spectral features were background-corrected and fitted with a mixture of Gaussian and Lorentzian fitting functions. The morphology and structural properties of the chromium oxide films were investigated with a Hitachi S-4100 model scanning electron microscope (SEM) and Siemens D5000 X-ray diffractometer. A Perkin Elmer Analyst 300 atomic absorption spectroscopy facility was used to probe Ringer's solution from the corrosion test for a determination of chromium ion concentrations released into the solution.

### 4.3. Electrochemical corrosion and Mott–Schottky analysis measurements

The electrochemical measurement on the chromium oxide-coated and -uncoated steel substrates was conducted in Ringer's solution at 37°C using a VoltaLab 40 consisting of a PGZ 301 potentiostat and a three-electrode electrochemical cell. The chemical composition of Ringer's solution used was as follows: NaCl (6.5 g l^−1^), KCl (0.42 g l^−1^), CaCl_2_ (0.25 g l^−1^) and NaHCO_3_ (0.20 g l^−1^). For the electrochemical test, the deposition times for the coatings were varied beyond 120 min for some samples to achieve thicknesses that are approximately equal to each other to guarantee a consistent comparison of the corrosion performance of the coated samples in Ringer's solutions. Chromium oxide-coated samples of thicknesses in the range of 210–220 nm were used in the investigation. The sample surface was used as the working electrode, while the reference and counter electrodes were a saturated calomel electrode (SCE) and platinum wire, respectively. Potentiodynamic polarization and electrochemical impedance spectroscopy (EIS) methods were employed in the corrosion investigation. Prior to the corrosion test, an open circuit potential measurement was performed for 2 h to enable the system to reach a steady state, thereby allowing the corrosion potential to stabilize before the experiment. The potentiodynamic polarization involved a polarization of the samples from −1000 mV to 1000 mV at the rate of 2 mV s^−1^ and the EIS measurements were conducted at a frequency range of 10 mHz to 40 kHz and an AC amplitude of 20 mV. The properties of the passive films formed on the surface of the samples were investigated with Mott–Schottky analysis. The samples were polarized from −1000 mV to +700 mV with successive steps of 50 mV.

### 4.4. Substrate plastic straining adhesion test

Each substrate for tensile testing was machined into a tensile ‘dog bone’ shape before coating and measured 120 × 20 mm with a thickness of 1 mm. The chromium oxide-coated dog bone samples (as-prepared films and films soaked in Ringer's solution) were subjected to an increasing tensile strain of 3% and 5% to initiate cracks in the films and to determine the strain at which initial cracking occurred using an Instron 50 KN machine. As the steel substrate was deformed plastically in tension, the chromium oxide films developed cracks that are normal to the loading axis. The samples were further strained to 10, 15, 20 and 25% to determine the saturation crack spacing. The gauge sections of all the chromium oxide-coated dog bone-shaped samples subjected to tensile testing were cut and examined with a Hitachi S-4100 model SEM; image analysis computer software on the SEM was used to measure crack spacings before an analysis of the crack spacing statistics on the films was conducted. One hundred crack spacing measurements were taken for each of the samples, and the results were tabulated for statistical analysis. The strain at which the film crack starts to appear gives the measure of the tensile fracture strength of the film; this was determined using equation (2.5) and the interfacial adhesion between the substrate material and the thin film coating can then be calculated using equation (2.6).

## 5. Results and discussion

### 5.1. Raman, Fourier transform infrared and X-ray photoelectron spectroscopic investigations

**Figure 1.**
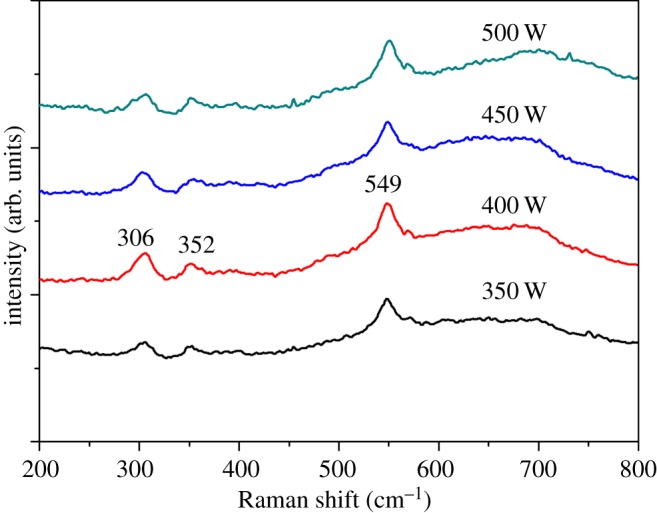
Raman spectra of chromium oxide coatings prepared at various deposition powers and a constant oxygen flow rate of 10 sccm.

The Raman spectra for chromium oxide coatings prepared at various forward powers showing three Raman peaks for each of the conditions are depicted in figure 1. An average of four to five Raman peaks have been reported by various authors for the Raman spectroscopic study of chromium oxide films in the literature. Khamlich *et al.* [34], in their investigation of the growth mechanism of α-Cr_2_O_3_ monodispersed particles prepared by aqueous chemical growth, observed four peaks: one $A_{1_{g}}$ mode of chromium oxide at 550 cm^−1^ and three other peaks at approximately 302, 349, 605 cm^−1^, which they attributed to the E_g_ mode. In a similar investigation by Barshilia & Rajam [26], the authors reported four Raman bands and identified the band centred at 544 cm^−1^ as $A_{1_{g}}$ symmetry and the other bands centred at 302, 349 and 605 cm^−1^ as the E_g_ symmetry of chromium oxide. Mougin *et al.* [35] reported further Raman peaks at 307, 524, 350, 551 and 610 cm^−1^ for chromium oxide obtained at ambient pressure. In our current investigation of chromium oxide films prepared at different deposition powers, we observed three Raman peaks that are associated with chromium oxide at approximately 306, 352 and 549 cm^−1^, with the most pronounced peak at 549 cm^−1^, as shown in figure 1. All the peaks correspond to one single phase of chromium oxide, i.e. Cr_2_O_3_ [26,29]. The little shift in the Raman peaks in this study compared to those reported in the literature is probably due to differences in the deposition methods and preparation conditions used in the production of the thin films.

**Figure 2.**
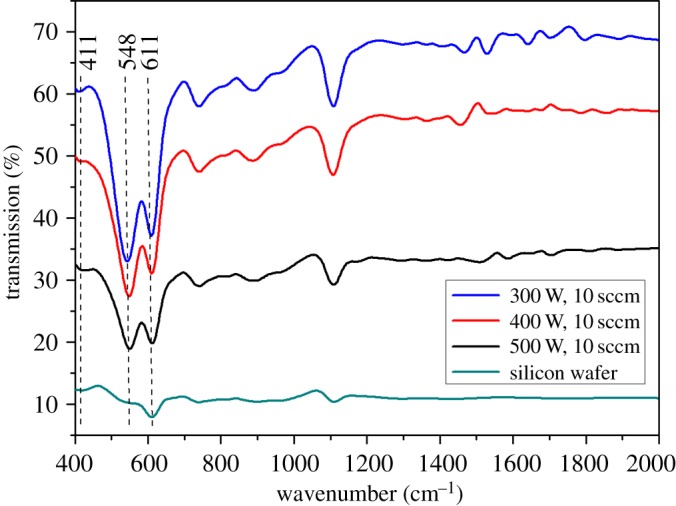
The FTIR spectra of chromium oxide coatings deposited on silicon wafer at various deposition powers and a constant oxygen flow rate of 10 sccm.

**Figure 3.**
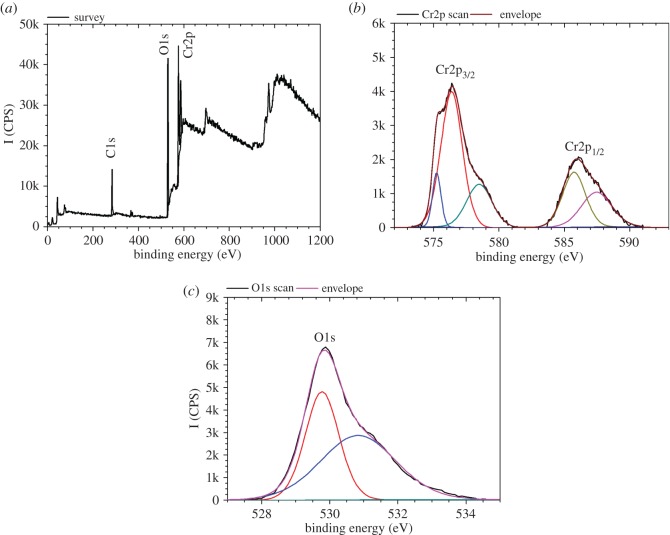
XPS scan of chromium oxide coating prepared at an oxygen flow rate of 10 sccm and deposition power of 500 W. (*a*) Survey scan, (*b*) Cr2p doublet and (*c*) O1s peak.

The FTIR spectra for chromium oxide coatings prepared at different deposition powers and an oxygen flow rate of 10 sccm on silicon wafer are shown in figure 2. Each spectrum is composed of peaks at 548 and 611 cm^−1^, both of which represent vibrational modes of Cr_2_O_3_ [36] and their intensity increases with an increase in the forward power used during the deposition of the films. The peak at 411 cm^−1^ was assigned to the E_u_ vibrational mode. Figure 3 shows the XPS spectrum for chromium oxide coating prepared at an oxygen flow rate of 10 sccm and a deposition power of 500 W. The survey scan (figure 3*a*) shows the presence of Cr, O and C elements in the sample. Highly resolved, fitted peaks for the Cr2p doublet and O1s are shown in figure 3*b,c*. The Cr2p_3/2_ spectrum showed peaks at binding energy positions of approximately 575.25, 576.40 and 578.46 eV. The peak at approximately 575.25 eV indicates a non-stoichiometric oxide phase between metallic chromium at 574.9 eV and the Cr_2_O_3_ peak located at 576.40 eV [29]. For the Cr2p_1/2_, binding energy peaks were obtained at 585.75–587.49 eV, indicating a non-stoichiometric oxide. The O1s spectrum showed a peak at a binding energy of 530.84 eV, which is usually associated with Cr_2_O_3_ [26,29].

### 5.2. Scanning electron microscopy and X-ray diffraction analysis

**Figure 4.**
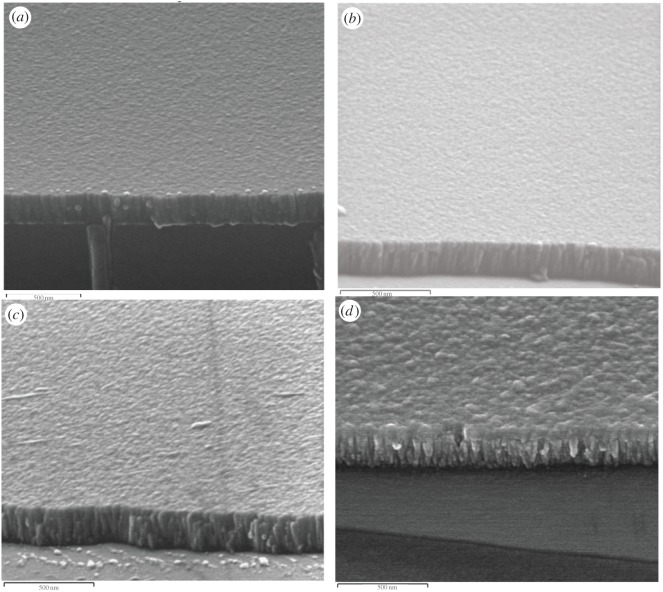
SEM images for chromium oxide coating prepared at a deposition power of 500 W and at an oxygen flow rate of (*a*) 4 sccm, (*b*) 6 sccm, (*c*) 8 sccm and (*d*) 10 sccm.

**Figure 5.**
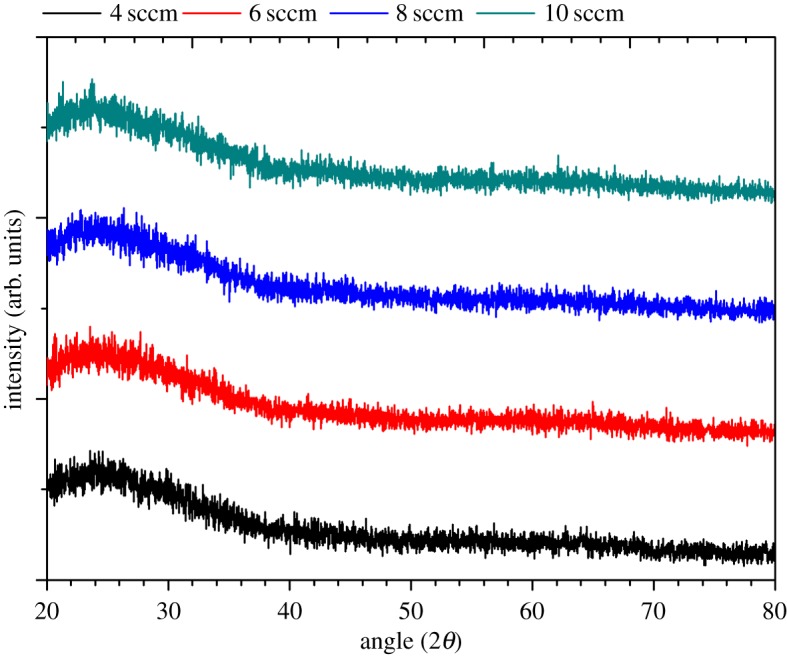
The XRD spectra for chromium oxide coatings prepared on glass slides at various oxygen flow rates and a constant deposition power of 500 W.

Cross-sectional images of the chromium oxide coatings prepared under various oxygen flow rates and a constant power of 500 W are depicted in figure 4. The SEM images indicate that the films have columnar structures but films prepared at lower oxygen flow rates appear to display a dense columnar structure. A dense or closely packed structure is a desirable surface feature for anti-corrosion films, which can help reduce the pathway through which aggressive ions such as chloride ions can diffuse and attack the substrate material. The results of the X-ray diffraction (XRD) analysis of the chromium oxide coatings are shown in figure 5. As can be seen from figure 5, no XRD peak was found in the scans, which suggests that the prepared films are predominately amorphous.

### 5.3. Potentiodynamic polarization

**Figure 6.**
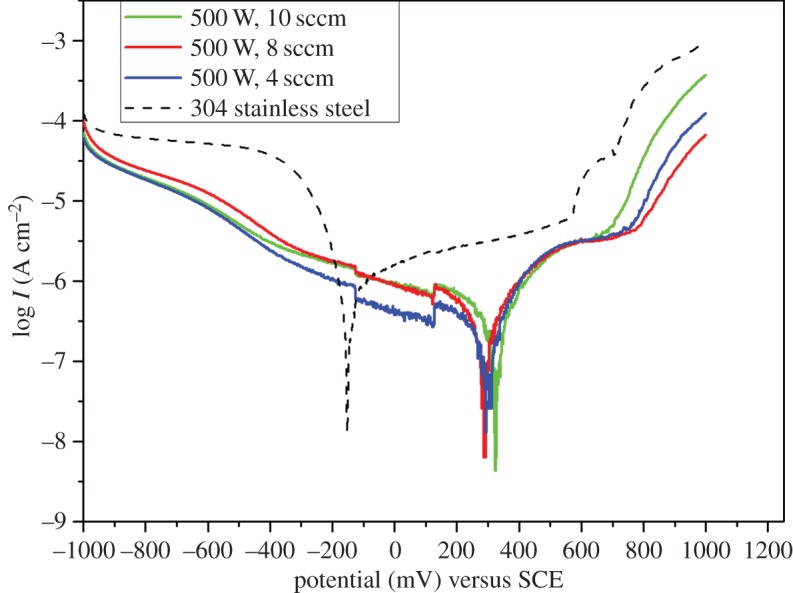
Potentiodynamic polarization curves of chromium oxide-coated and -uncoated stainless steel substrates in Ringer's solution at 37°C.

The Tafel plots obtained from the potentiodynamic polarization test for the corrosion of chromium oxide-coated and -uncoated steel in Ringer's solution at 37°C are presented in figure 6. The corrosion parameters (corrosion potential, corrosion current and polarization resistance) for both chromium oxide-coated and -uncoated stainless steel were determined from the Tafel extrapolation method and are presented in table 2. Tafel plots for chromium oxide-coated stainless steel showed a decrease in current density (*I*_corr_) as well as a positive shift in corrosion potential (*E*_corr_) with respect to the uncoated stainless steel substrate, which implies a lower corrosion susceptibility for the chromium oxide-coated substrates. Higher polarization resistance (*R*_p_) values were also observed for chromium oxide-coated samples. Furthermore, for all the chromium oxide coatings, the breakdown potentials were more positive compared to those of the uncoated stainless steel, which is an indication that the coatings have a better pitting corrosion resistance and possess the capability to protect the stainless steel in Ringer's solution. The chromium oxide coatings prepared under various oxygen flow rates showed a varying degree of protection of the stainless steel substrate, which is evident in the values of the corrosion parameters such as current density obtained from the Tafel extrapolation. The chromium oxide coating prepared at an oxygen flow rate of 4 sccm showed the least current density, followed by the 8 sccm sample, with the sample deposited at 10 sccm showing the highest current density among the coated samples, as illustrated in table 2. This trend in the coating performance is most probably due to a decrease in the compactness of the films with the increase in oxygen flow rates used during deposition, which was earlier observed during the SEM characterization of the samples.

**Table 2.** Corrosion parameters from potentiodynamic polarization for chromium oxide-coated and -uncoated 304 stainless steel in Ringer's solution at 37°C.

sample	*E*_corr_ (mV) versus SCE	*I*_corr_ (µA)	*R_p_* (KΩ)
304 stainless steel	−156	0.538	45.29
chromium oxide-coated at 4 sccm	294	0.169	175.95
chromium oxide-coated at 8 sccm	284	0.319	130.58
chromium oxide-coated at 10 sccm	321	0.409	126.30

**Figure 7.**
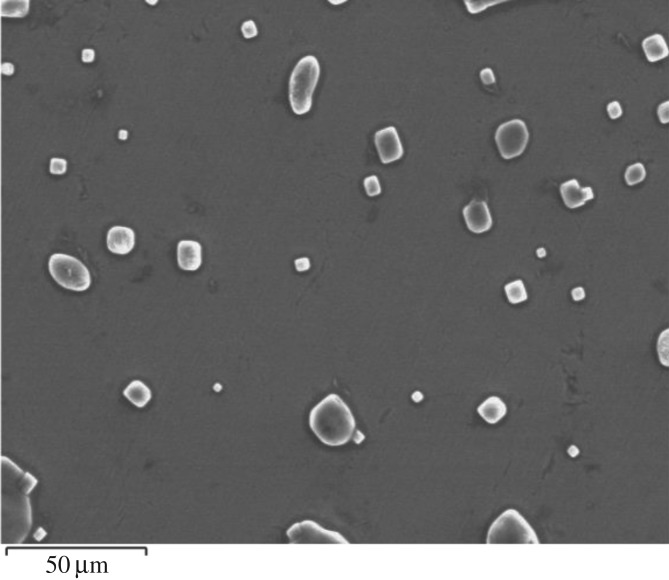
An SEM image of chromium oxide coating in the background (intact) after corrosion testing in Ringer's solution at 37°C showing crystallites from Ringer's salt solution.

The potentiodynamic polarization results show that chromium oxide coatings not only possess a better electrochemical stability in solutions such as PBS solution [28] and saline solution [26,29,30] as previously reported in the literature, but they also have superior electrochemical stability, compared to bare uncoated stainless steel in Ringer's solution. This makes them a promising candidate material for medical implant applications. Figure 7 shows a typical SEM image of a chromium oxide-coated sample after the potentiodynamic polarization test in Ringer's solution. As can be seen from the SEM image, the coated sample showed a good resistance to corrosion with no sign of pitting corrosion, but with crystallites from Ringer's solution deposited on the surface.

### 5.4. Electrochemical impedance spectroscopic measurements

**Figure 8.**
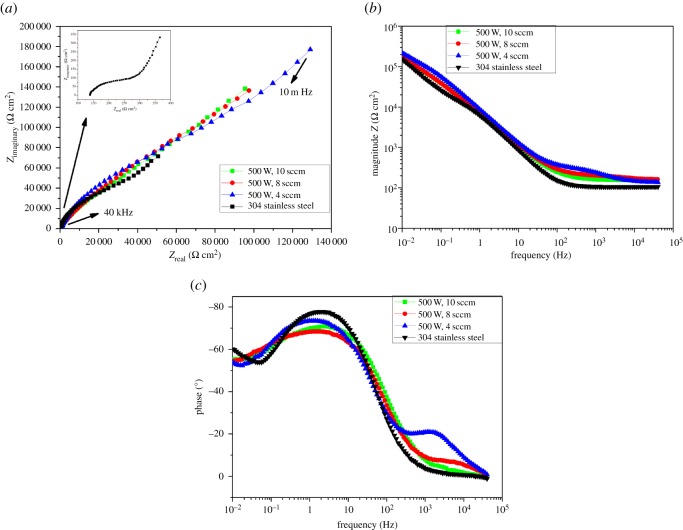
EIS plots for chromium oxide-coated and -uncoated stainless steel in Ringer's solution at 37°C. (*a*) Nyquist plots, (*b*) Bode plots (magnitude versus frequency) and (*c*) Bode plots (phase angle versus frequency).

**Figure 9.**
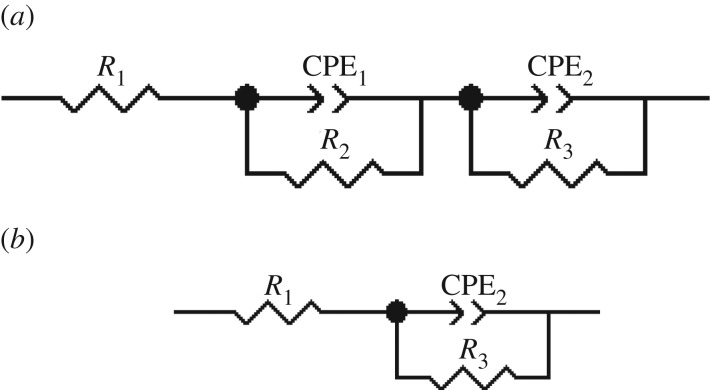
Equivalent circuit model used for fitting of experimental data of chromium oxide-coated stainless steel (*a*) and uncoated stainless steel substrates (*b*) exposed to Ringer's solution at 37°C.

The impedance spectra obtained from EIS measurements for the corrosion of chromium oxide-coated and -uncoated stainless steel substrates in Ringer's solution at 37°C are shown as Nyquist and Bode plots in figure 8*a–c*. The data were analysed by fitting them to an equivalent circuit model shown in figure 9 using the Z view software, and a summary of the results is presented in table 3. The quality of the fitting was judged by the *χ*^2^-value that was less than 10^−3^ and by the error percentage distribution between the experimental and simulated data. The capacitance behaviour of the samples was simulated with the constant phase element (CPE) instead of pure capacitors to account for the inhomogeneous state of the sample surfaces. In the equivalent circuit in figure 9, *R*_1_ represents the solution resistance, *R*_2_ and CPE_1_ are the coating resistance and capacitance (for the outer part of the coating system), representing the electrochemical behaviour at high frequencies. The circuit elements *R*_3_ and CPE_2_ are the interfacial oxide resistance and capacitance of the coating material (for the inner part of the coating system), which account for the electrochemical response at low frequencies. CPE–*P* is the exponent index, which represents the deviation of the capacitance of the passive film from the ideal behaviour. When CPE–*P* = 1, it implies pure capacitive behaviour of the CPE and CPE–*P* = 0 indicates resistive behaviour.

**Table 3.** Electrochemical impedance parameters for chromium oxide-coated and -uncoated stainless steel exposed to Ringer's solution obtained using the equivalent circuit shown in figure 9.

	*R*_1_	$\text{CP}{\text{E}}_{1}$		*R*_2_	$\text{CP}{\text{E}}_{2}$		$R_{3}$
sample	($\Omega .{\text{cm}}^{2}$)	$\left(\text{c}{\text{m}}^{-2}S^{-n}\Omega \right)$	$\text{CP}{\text{E}}_{1}-P$	($\Omega .{\text{cm}}^{2}$)	$\left(\text{c}{\text{m}}^{-2}S^{-n}\Omega \right)$	$\text{CP}{\text{E}}_{2}-P$	$\left(\Omega .\text{c}{\text{m}}^{2}\right)$
304 stainless steel	198.6	—	—	—	2.64 × 10^−5^	0.89	0.13 × 10^6^
chromium oxide-coated at 4 sccm	201.1	3.72 × 10^−5^	0.89	40424	6.75 × 10^−5^	0.93	0.95 × 10^6^
chromium oxide-coated at 8 sccm	178.4	1.24 × 10^−4^	0.76	16326	4.64 × 10^−5^	0.90	0.80 × 10^6^
chromium oxide-coated at 10 sccm	151.8	6.74 × 10^−5^	0.81	38855	5.52 × 10^−5^	0.85	0.66 × 10^6^

As can be seen from the Nyquist plots in figure 8*a*, a significant increase in the impedance value was observed for stainless steel coated with chromium oxide coatings compared to bare stainless steel, which is an indication of an increase in corrosion resistance of the samples. The improvement in the corrosion resistance is also evident in the Bode plots (magnitude versus frequency) shown in figure 8*b,* where the impedance values of all chromium oxide coatings lie above that of bare stainless steel. The chromium oxide-coated sample prepared at an oxygen flow rate of 4 sccm showed the highest impedance value (i.e. the highest corrosion resistance), which is in agreement with the results from the potentiodynamic polarization technique where the same sample exhibited the least corrosion current density and highest polarization resistance values. The values of *R*_3_ obtained for chromium oxide prepared under various conditions were much higher than *R*_2_ values, which suggest that the inner configuration of the coating is likely to be responsible for corrosion protection provided on the stainless steel substrate by chromium oxide coatings.

The Bode plots shown in figure 8*c* indicate that chromium oxide-coated and -uncoated stainless steel exhibited phase angles near −60° at low frequencies and shifted to a phase angle close to −90° at intermediate frequencies. This implies a nearly capacitive behaviour, which is typical of a passive material.

### 5.5. Mott–Schottky analysis

**Figure 10.**
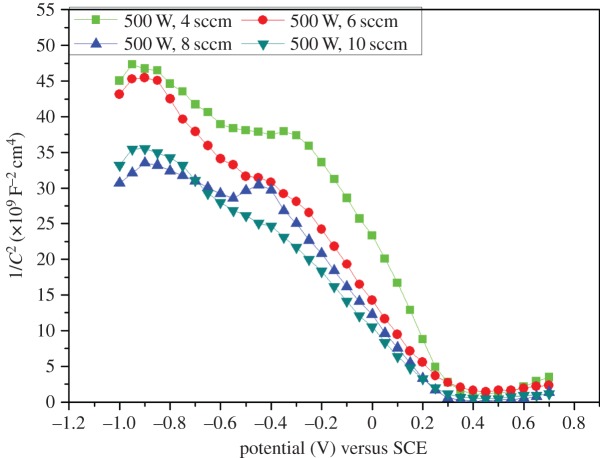
Mott–Schottky plots of chromium oxide coatings prepared at different oxygen flow rates in Ringer's solution at 37°C.

**Figure 11.**
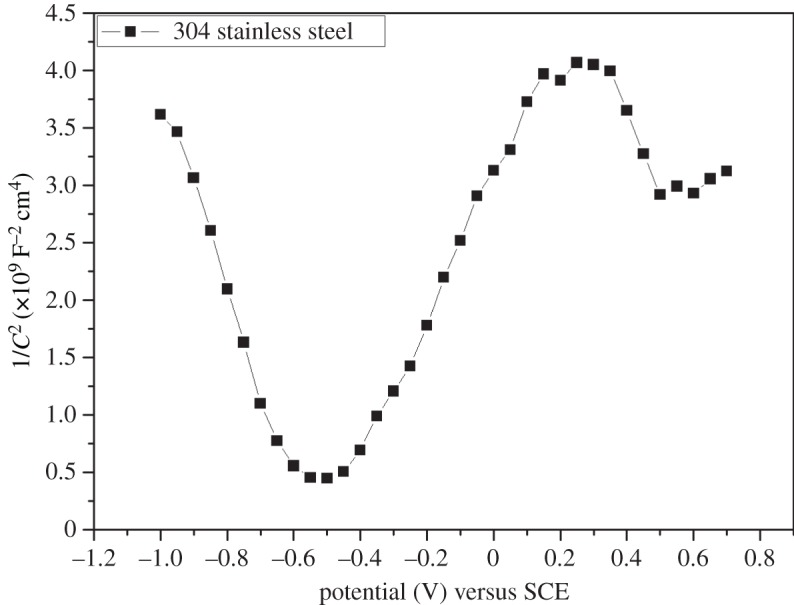
Mott–Schottky plot of uncoated stainless steel in Ringer's solution at 37°C.

The Mott–Schottky plots for chromium oxide-coated and -uncoated steel exposed to Ringer's solution at 37°C are shown in figures 10 and 11. The chromium oxide coatings prepared using various deposition conditions gave Mott–Schottky plots that are all negative, which implies that the oxide layers exhibit a *p*-type semiconductor property. The Mott–Schottky plot for uncoated stainless steel showed that it possesses a duplex structure, i.e. *n*-type and *p*-type semiconductor behaviour. The *n*-type semiconductor behaviour exhibited by the passive films on stainless steel can be attributed to the iron-rich layer, while the *p*-type semiconductivity of the passive film is the result of the presence of chromium in the inner layer [29]. The Mott–Schottky plots for chromium oxide coatings show that increasing the oxygen flow rate during deposition led to a decrease in the slope of the Mott–Schottky straight line and a corresponding increase in the acceptor defect density. The lower the oxygen flow rate during deposition, the lower the acceptor defect density obtained from the capacitance measurements. The lowest acceptor defect density was observed for chromium oxide coatings prepared at an oxygen flow rate of 4 sccm. The structure of the film is likely to have influenced the nature of the passive film formed on the coated samples, with denser films showing a lower defect density.

The uncoated stainless steel sample exhibited a higher defect density when compared with the chromium oxide-coated samples, which is an indication of a higher disorder defect density in the passive native films on uncoated steel, as illustrated in table 4. The lower acceptor defect densities of the passive film formed on chromium oxide coatings reflect the better passivation potential of chromium oxide-coated substrates compared to the bare stainless steel, which exhibited a highly defective passive film.

**Table 4.** Mott–Schottky results for chromium oxide-coated and -uncoated stainless steel in Ringer's at 37°C.

sample	defect type	*N* (cm^−3^)	*E*_fb_ (mV) versus SCE
304 stainless steel	*n*-type	8.87 × 10^20^	−520
304 stainless steel	*p*-type	6.41 × 10^20^	−560
chromium oxide-coated at 4 scm	*p*-type	8.58 × 10^19^	320
chromium oxide-coated at 6 scm	*p*-type	1.24 × 10^20^	310
chromium oxide-coated at 8 sccm	*p*-type	1.34 × 10^20^	300
chromium oxide-coated at 10 sccm	*p*-type	1.74 × 10^20^	295

The Mott–Schottky result is supported by our earlier corrosion experiments discussed above based on potentiodynamic polarization and EIS where chromium oxide-coated samples showed a better corrosion resistance when compared to uncoated steel substrate. A similar observation was previously reported in a Mott–Schottky analysis on chromium oxide-coated and -uncoated stainless steel substrates tested in saline solution by the present authors [29].

### 5.6. Ion release measurements

**Figure 12.**
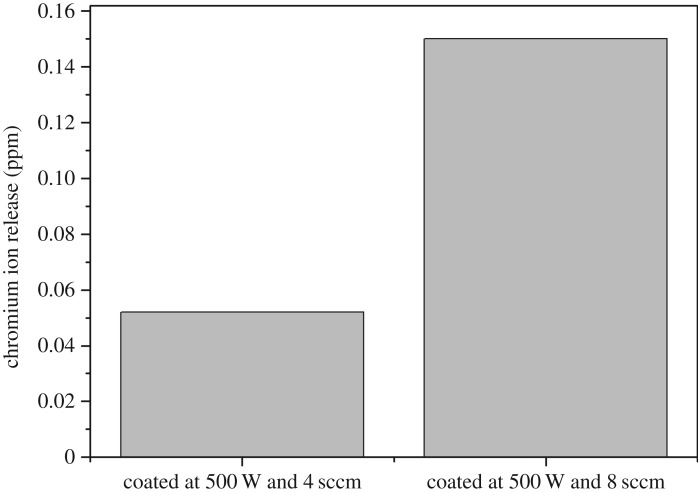
Chromium ion release from chromium oxide coatings prepared at oxygen flow rates of 4 sscm and 8 sccm and a deposition power of 500 W exposed to Ringer's solution at 37°C, for samples polarized to 1500 mV during electrochemical testing.

Ringer's solution used during the electrochemical corrosion testing of the chromium oxide coatings prepared at various deposition conditions was probed for the presence of chromium ions. The results of our atomic absorption spectroscopic measurement for chromium oxide-coated samples polarized from −1000 to 1500 mV in Ringer's solution at 37°C are shown in figure 12. We observed zero absorbance values for the solutions taken from samples exposed to open circuit potential measurements or the lower corrosion potentials expected to be experienced by implants in the human body. This implies that the concentration of chromium ions, if any, released into the solution is below the detection limit of the equipment at the ppm level. However, negligible chromium ion concentrations were observed at extreme potential values up to 1500 mV.

### 5.7. Substrate-straining adhesion measurements

**Figure 13.**
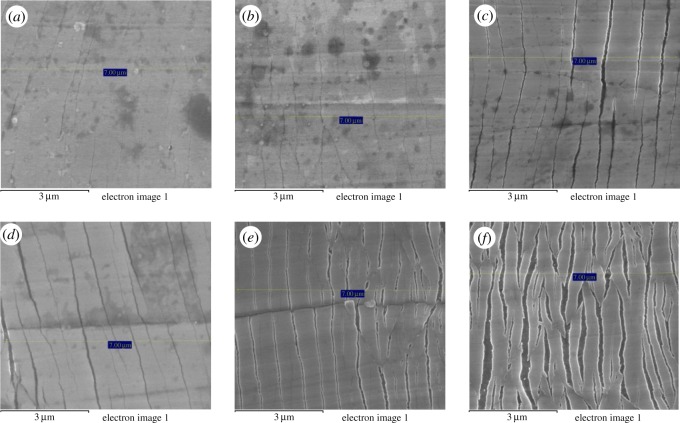
(*a*) Typical SEM image of chromium oxide film on stainless steel substrate strained to 3% showing initial crack formation. SEM images of (*b*) sample strained to 5%; (*c*) sample strained to 10%; (*d*) sample strained to 15%; (*e*) observed saturation crack spacing and film delamination for sample strained to 20% and (*f*) sample strained to 25% with signs of film defoliation.

**Figure 14.**
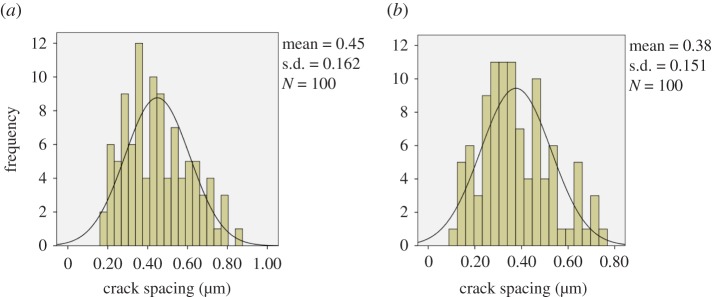
(*a*) Typical measured crack spacing on tensile-strained chromium oxide films on steel substrates at 20%. (*b*) Typical measured crack spacing on tensile-strained chromium oxide films on steel substrates at 25%.

One hundred crack spacing measurements were taken for each of the samples strained to 5, 10, 15, 20 and 25%. Typical SEM images and the crack frequency distribution observed at various strains for the as-prepared chromium oxide thin films are shown in figures 13*a–f* and 14*a,b*. The probability plots for fitting the observed crack spacing distributions are as shown in figure 17.

**Figure 15.**
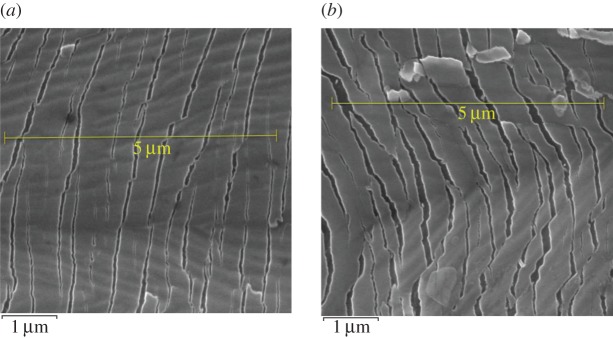
(*a*) Typical observed saturation crack spacing and film delamination for coatings soaked in Ringer's solution for one month and strained to 20%. (*b*) Samples soaked in Ringer's solution for one month and strained to 25% with signs of film defoliation.

**Figure 16.**
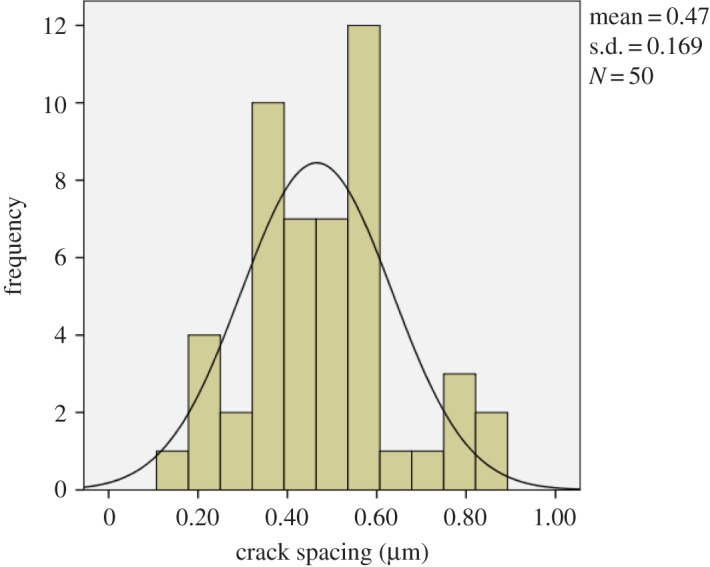
Crack spacing frequency distribution of chromium oxide films in Ringer's solution for one month strained to 20%.

**Figure 17.**
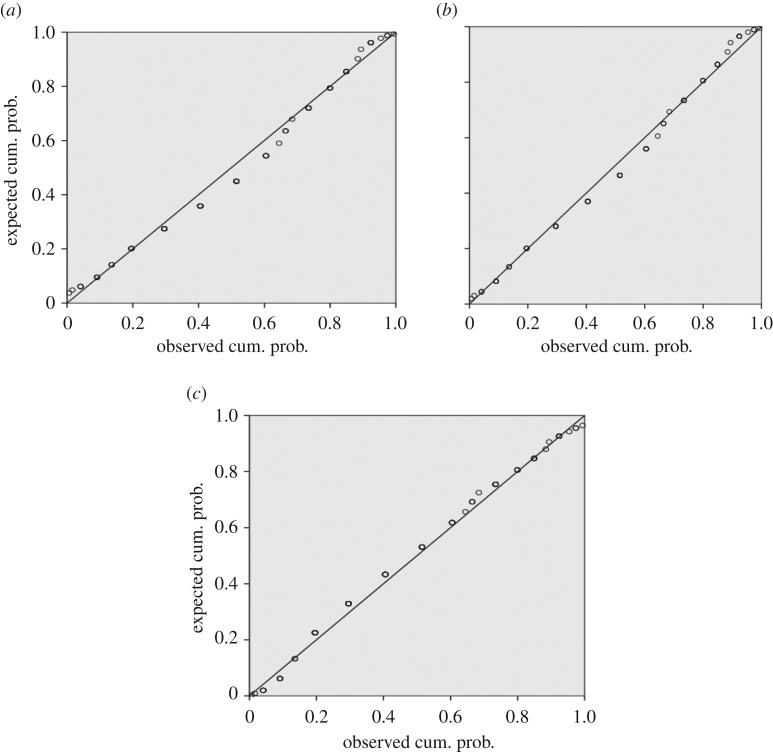
(*a*) A fitting of the observed spacing distribution at saturation in the chromium oxide films to the normal probability distribution. (*b*) A fitting of the observed spacing distribution at saturation in chromium oxide films to the Weibull probability distribution. (*c*) A fitting of the observed spacing distribution at saturation in chromium oxide films to the lognormal probability distribution.

**Figure 18.**
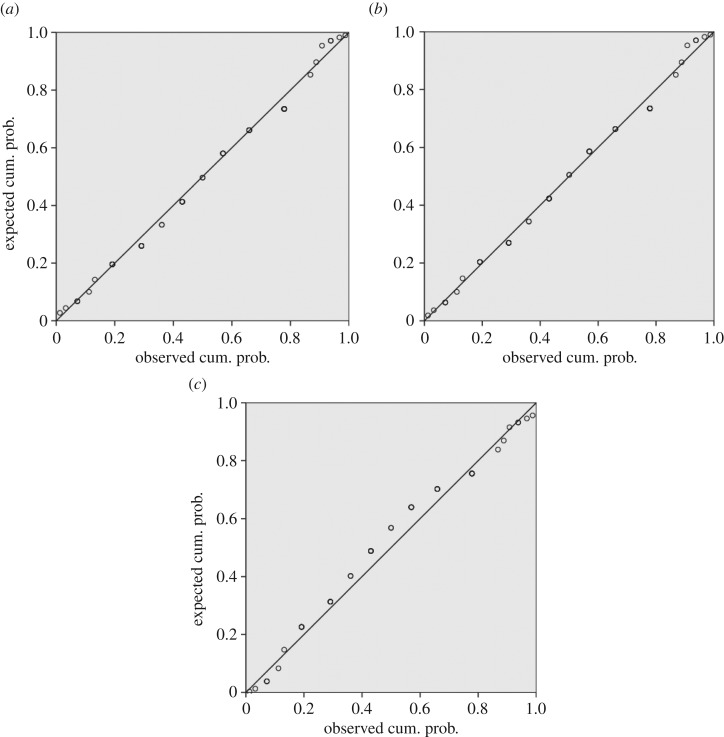
(*a*) Observed spacing distribution at saturation in the chromium oxide films soaked in Ringer's solution fitted to the normal probability distribution; (*b*) observed spacing distribution at saturation in the chromium oxide films soaked in Ringer's solution fitted to the Weibull probability distribution; (*c*) observed spacing distribution at saturation in the chromium oxide films soaked in Ringer's solution fitted to the lognormal probability distribution.

**Figure 19.**
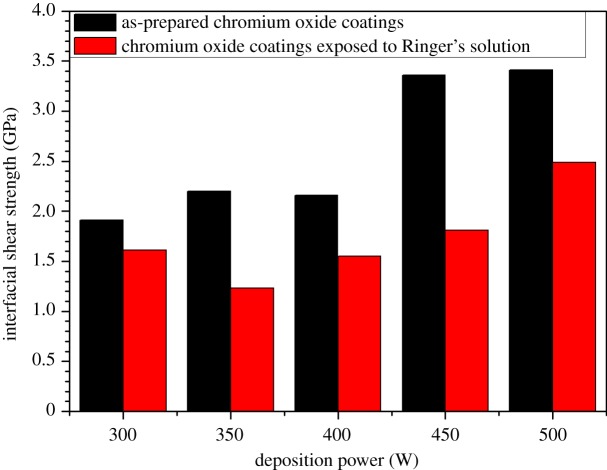
The change in interfacial shear strength for chromium oxide coatings exposed to Ringer's solution for a period of one month.

The SEM images and crack frequency distribution observed at various strains for the films soaked in Ringer's solution for the period of one month are shown in figures 15*a,b* and 16. The crack spacing measurements and SEM images suggest that saturation occurred at 25% tensile strain, with exfoliation and delamination of the films witnessed at this stage, as depicted in figures 13*f* and 15*b*. The crack spacing distribution for the tensile-strained chromium oxide thin films (as-prepared films) on stainless steel was found to fit closely to the lognormal distribution function among various probability distribution functions considered, as shown in figure 17*a–c,* using the statistical software package SPPS. The mean spacing was determined from this function and used in the relationship shown in equation (2.6) to determine the interfacial shear strength of the prepared films. For the films soaked in Ringer's solution, the crack spacing distribution for the samples prepared at forward powers of 300 and 500 W was found to fit closely to the lognormal distribution, while films prepared at forward powers of 350, 400 and 450 W changed to the Weibull distribution functions, as shown in figure 18*a–c*. A summary of the adhesion measurement results for the as-prepared films and films soaked in Ringer's solution for one month is shown in figure 19.

As can be seen from figure 19, a decrease in interfacial shear strength was observed for the films soaked in Ringer's solution for the period of one month when compared to the as-prepared films. Ogwu *et al.* [37,38] and Chandra *et al.* [39] have previously investigated the influence of biological fluid on the crack spacing distribution and the adhesion strength of Si-DLC films prepared on steel substrates. The authors observed lower interfacial shear strength for films exposed to biological fluids or saline solution when compared to as-prepared samples.

They proposed that the presence of nanoporosities in the DLC/Si-DLC films deposited on steel substrate could have acted as a source for crack advancement in the films during the adhesion test [37–39]. The authors suggested that on the exposure of the films to biological fluids prior to the substrate-straining test, these defects or nanoporosities aid the penetration of fluids at the interface between the films and the substrate, which has an effect on the adhesion and residual stress distribution in the films. In our current investigation, the above mechanism is suggested as being responsible for the decrease in adhesion strength of films soaked in Ringer's solution. We observed a crack spacing distribution that fitted closely to the lognormal distribution for all of our as-prepared samples. However, on exposure to Ringer's solution for one month, a change to the Weibull distribution function was observed with the exception of films prepared at 500 and 300 W, which still fitted closely to the lognormal function. This change is most probably due to a variation in the microstructure of the coatings and nanoporosities present within the chromium oxide films, resulting from the deposition conditions used during the deposition process. The interfacial strength of the prepared chromium oxide coatings obtained in this study is comparable to those reported for DLC and Si-DLC coatings by various authors in the literature [37–39].

## 6. Conclusion

The corrosion behaviour of chromium oxide coatings prepared by reactive magnetron sputtering has been investigated at 37°C in Ringer's solution. The corrosion results based on potentiodynamic polarization and EIS measurements showed an improvement in corrosion resistance for chromium oxide-coated stainless steel samples compared to the bare stainless steel substrate. The corrosion current density for chromium oxide-coated stainless steel is much lower than for uncoated stainless steel, suggesting the presence of a higher resistant passive film on the coatings. The Mott–Schottky analysis revealed that the chromium oxide-coated stainless steel samples possess a *p*-type semiconductor behaviour with a lower defect density with respect to bare stainless steel, which exhibited highly defective passive films characterized by the duplex structure (*n*-type and *p*-type semiconductor).

The chromium oxide coating prepared at an oxygen flow rate of 4 sccm revealed the lowest defect density and the best corrosion resistance in Ringer's solution at 37°C. The as-prepared chromium oxide films showed a good adhesion to the steel substrate, but a reduction in the interfacial adhesion value was observed for the coatings when they were exposed to Ringer's solution prior to the adhesion test. The presence of nanopores or pinholes in the coating is thought to have contributed to the change in adhesion strength as these pinholes can act as a pathway for fluid penetration unto the substrate, thereby weakening the coating/substrate system. The interfacial strength of the prepared chromium oxide coatings obtained in this study is comparable to that reported for Si-DLC films. The ion release results from the atomic absorption measurements indicate that there was no chromium ions released from the chromium oxide-coated samples into Ringer's solution at the ppm level, for samples tested under open circuit electrochemical testing conditions and at the relatively lower corrosion potentials expected on implants in the human body. The corrosion and adhesion results as well as the negligible chromium ion release into Ringer's solution open the door to progressing to evaluating the immune cell activation and other biocompatibility tests required for orthopaedic and other possible medical implant applications of chromium oxide coatings. Chromium ion release into body fluids *in vivo* is still an outstanding issue in patients with orthopaedic implants.

## Data accessibility

All of the data in this investigation have been reported in the paper and are freely available.
